# The Effect of Chloride and Sulfate Ions on the Adsorption of Cd^**2+**^ on Clay and Sandy Loam Egyptian Soils

**DOI:** 10.1155/2014/806252

**Published:** 2014-04-02

**Authors:** Mohamed E. EL-Hefnawy, Elmetwaly M. Selim, Faiz F. Assaad, Ali I. Ismail

**Affiliations:** ^1^Chemistry Department, Rabigh College of Sciences and Arts, King Abdulaziz University, P.O. Box 344, Rabigh 21911, Saudi Arabia; ^2^Department of Chemistry, Faculty of Science, Tanta University, Tanta 31527, Egypt; ^3^Biological Sciences Department, Rabigh College of Science and Arts, King Abdulaziz University, P.O. Box 344, Rabigh 21911, Saudi Arabia; ^4^Soils and Water Use Department, Agricultural and Biological Division, National Research Centre, 33 Al Behoos Street, P.O. Box 12622, Dokki, Cairo, Egypt

## Abstract

Adsorption of Cd^2+^ on two types of Egyptian soils: clay (alluvial) and sandy loam (calcareous), was studied. Effect of changing the matrix electrolyte type and concentration was used to mimic the natural soil salts. Kinetics and thermodynamic parameters of the adsorption were calculated at two different electrolyte concentrations: 0.05 N and 0.15 N. The adsorption was described by Langmuir and Freundlich isotherms. Results showed that lower concentration of the NaCl or Na_2_SO_4_ electrolytes (0.05 N) had higher adsorption capacity. Also, the maximum adsorption of cadmium when using sulfate counter ion is about two to three times higher than that when using chloride (544 **μ**g/g for alluvial soil and 170 **μ**g/g for calcareous soil when using 0.05 N). Using NaCl as matrix electrolyte, Freundlich isotherms showed bi-linear fits that probably mean a two energy level adsorption. This might be explained by either the competition of Cd^2+^ with Na^+^ or its complexation with Cl^−^.

## 1. Introduction

Soil is known to hold heavy metals due to the existence of functional groups on the humic acid and other components of the soil [1–7]. Heavy metals of special interest have been studied as an environmental target on soil [2, 3]. In Egypt different types of soils were used to determine their capacity to hold several types of metals [8–16]. Addition of salts to the soil has been used to change the charge and general environment of the soil and to alter the adsorption characteristics toward heavy metals [17–21]. Cadmium is a toxic metal occurring in the environment naturally and coming from different sources mainly from industrial and agricultural sources. The mobility mechanism of heavy metals is an important parameter to understand the strength of the adsorption of the metal ions by the soil components [22]. The occurrence of heavy metals in the soils can theoretically include many fractions of them, free, complexed ions in pore water, or adsorbed “bound” on soil component surfaces. Also, they can be precipitated or coprecipitated as chemical compounds (hydroxides, carbonates, and sulfides) [23]. It is thought that the binding between cadmium ions and corresponding ligands affects their chemical behavior and toxicity in soil environments [24–28]. Cadmium is known to have a nephrotoxicity, initially causing kidney tubular damage which may lead to bone damage [24–28].

A recent theoretical study done by Chang Chien et al. suggested that addition of chloride and sulfate leads to an increase in the ability of soil to adhere more cadmium ions through enhancing the complexation with the anions [29]. The mechanism of competitive complexation and/or chelation between Cd^2+^ free cations and preferential concentrations of Cl^−^, SO_4_^2−^ anions results in significantly altering the adsorption behavior [29]. Addition of sulfate to Swiss chard showed an increase in the adsorption of cadmium ions [20]. Chloride was also used to increase the cadmium uptake, however, with less effect than sulfate [30].

The objective of this study is to investigate the adsorption of the cadmium ions on two Egyptian soils, alluvial and calcareous. The study will focus on the effect of addition of different types and concentration of electrolytes (like Cl^−^ and SO_4_^2−^). The aspects of cadmium mobility and the thermodynamic free energy of adsorption will be determined. These together might extend our knowledge about better understanding of the cadmium-soil binding and the effect of chloride and sulfate on this binding considering their competitive effect on the adsorption sites or on cadmium ions.

## 2. Materials and Methods

**Figure 1:**
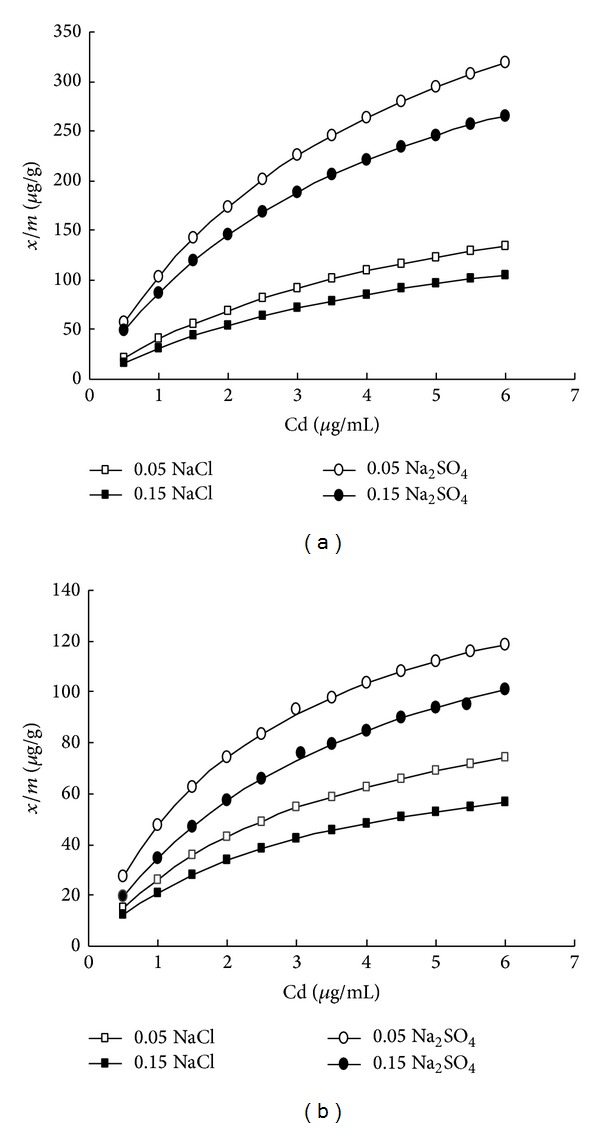
Cadmium adsorption isotherms by (a) alluvial soil and (b) calcareous soil, at different concentrations of NaCl and Na_2_SO_4_ as electrolyte matrices. Empty circles are for 0.05 N, solid circles for 0.15 N NaCl, empty squares for 0.05 N Na_2_SO_4_, and solid squares for 0.15 N Na_2_SO_4_.

The chemical, physical, and mineralogical properties of the two soils used in this study, alluvial and calcareous, were summarized in Table 1. The adsorption isotherms were conducted at pH = 7.8. Duplicate samples of each soil were applied. To 100 mL polyethylene tube, 2.00 g of soil sample and 50 mL of 0.05 NaCl were added. Appropriate volume of stock solution containing 500 mg Cd^2+^/L was added to obtain cadmium concentrations of 1–15 *μ*g/mL to each tube. The tubes were shaken in an end-over shaker (speed = 50 rpm) for 24 hours at room temperature. The pH was adjusted three times to be 7.8 during this period by adding very small amount of 0.1 M HCl or 0.1 M NaOH. The slurry was separated using a centrifuge (speed = 500 rpm, 2 minutes). The supernatant was decanted and used to calculate the amount of remaining cadmium. The adsorbed cadmium was calculated by the difference between the concentrations of Cd^2+^ in the original solution and in the equilibrium solution (decanted solution). The cadmium concentration was measured by atomic absorption spectroscopy (Shimadzu AA-6200) and the results are shown in Figure 1. The effect of changing the concentration of NaCl was studied by performing cadmium adsorption isotherms, with addition of 50 mL of 0.15 N NaCl instead of 0.05 N (see Figure 1). Also, 0.05 N and 0.15 N of Na_2_SO_4_ were used, instead of NaCl with same experimental procedure as above, to study the counter ion effect.

## 3. Results and Discussion

### 3.1. Cadmium Adsorption Isotherms

Two different types of soils from different regions of Egypt were selected for this study: alluvial and calcareous soils. The choice of these soils is for their big differences in the chemical and mineralogical properties, mainly in texture (% clay = 56.3 for alluvial soil and 11.7 for calcareous soil) and specific surface area (233.8 m^2^/g for alluvial and 87.6 m^2^/g for calcareous soil), as shown in Table 1. The big change in the characteristics between these soils may directly or indirectly affect the ability to adsorb heavy metals (or more specifically Cd^2+^ for the interest of this study).

The wide range of cadmium ion concentration used in this study produced curvilinear adsorption isotherms as shown in Figure 1(a) for adsorption of cadmium ion on alluvial soil and Figure 1(b) on calcareous soil. The results showed that the alluvial soil has 2 to 3 times higher adsorption ability toward cadmium ions than calcareous soil. In general, the lower the concentration of the counter ion (sulfate or chloride), the higher the adsorption capacity, of either soil used, to the cadmium ions. Also, the existence of sulfate ions significantly increases the adsorption ability of the soils toward cadmium ions compared to the chloride counter ion. To quantitatively determine the adsorption capacity, it is better to use the “Cd^2+^ adsorption Isotherm Gradients” as a reference. This reference can be calculated from the first linear parts of Figure 1, which will be used to obtain the main parameters for Langmuir and Freundlich isotherms, using (1) and (2). The values of the extracted parameters were summarized for the two soils with different electrolytes in Table 2. The maximum adsorption parameter, “*a*,” in Langmuir isotherm for alluvial soil (0.05 N Na_2_SO_4_) shows a value of 544 *μ*g/g whereas it is 170 *μ*g/g for calcareous soil (0.05 N Na_2_SO_4_).

**Figure 2:**
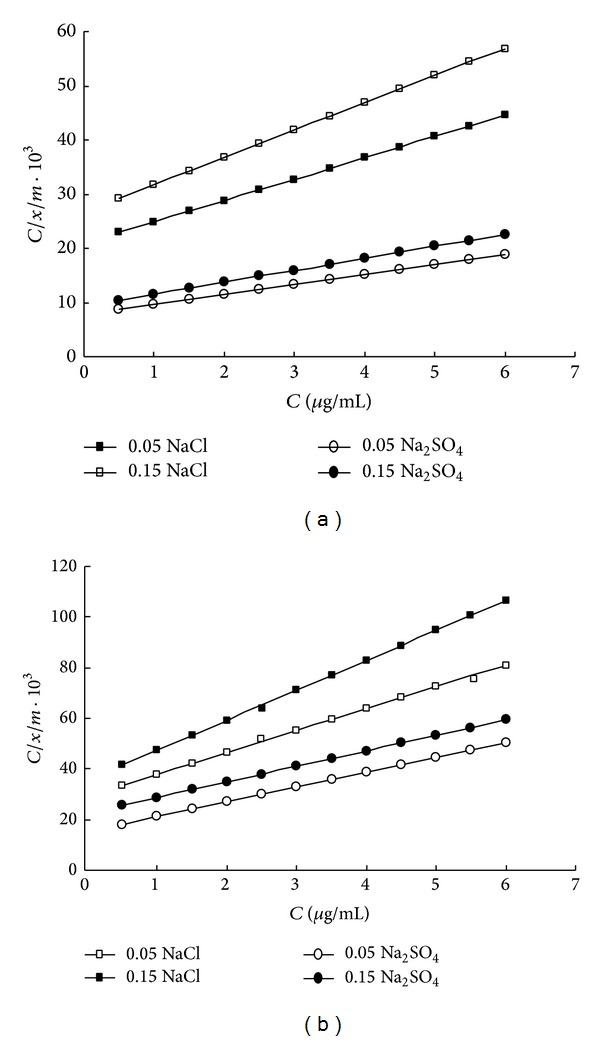
Langmuir isotherm adsorption of (a) alluvial soil and (b) calcareous soil, toward Cd^2+^, at different concentrations of NaCl and Na_2_SO_4_ as electrolyte matrices.

The adsorption of Cd^2+^ on these soils was fit into the linear form of Langmuir equation:

(1) $$\begin{array}{c} \frac{C}{\left(x/m\right)}=\frac{1}{\left(a\cdot b\right)}+\left[\frac{C}{a}\right], \end{array}$$

where (*x*/*m*) is the amount of Cd^2+^ adsorbed by unit weight soil (**μ**g/g), *C* is the equilibrium Cd^2+^ concentration in solution (**μ**g/mL), “*a*” is the Langmuir adsorption maximum (**μ**g/g), and “*b*” is the Langmuir “bonding term” related to bonding energy. To determine the maximum adsorption “*a*” and the bonding term “*b*” for the two soils under different salt matrix conditions and different ionic strengths, *C*/(*x*/*m*) was plotted against *C* resulting in straight lines. The fitted lines of the Langmuir isotherms are shown in Figure 2.

**Figure 3:**
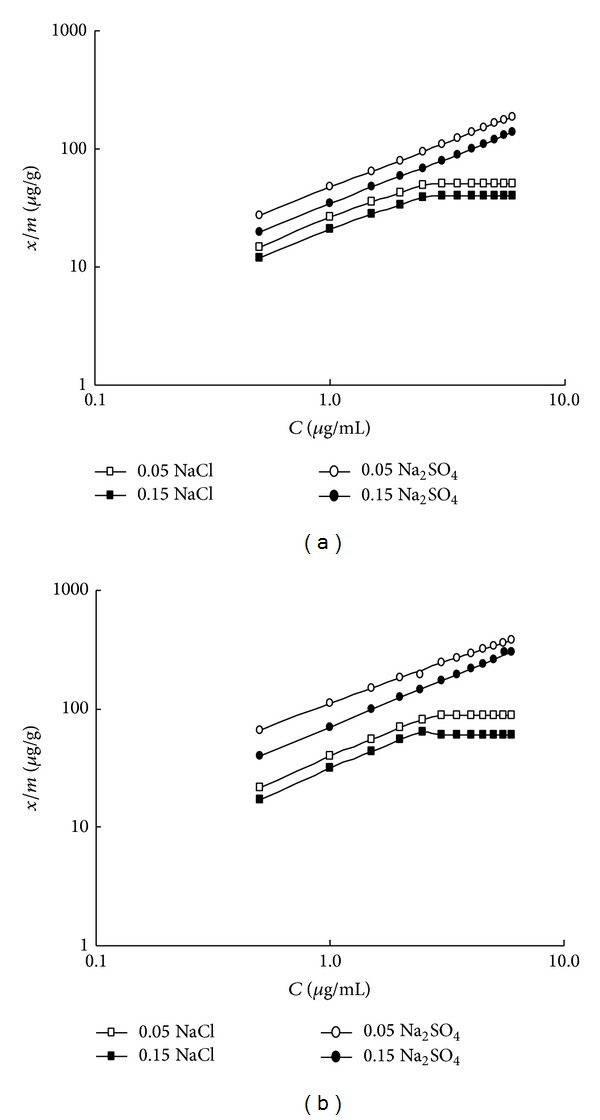
Freundlich plots of cadmium adsorption by (a) alluvial soil and (b) calcareous soil, at different concentrations of NaCl and Na_2_SO_4_ as electrolyte matrices.

Also, Freundlich equation

(2) $$\begin{array}{c} \text{Log}\left(\frac{x}{m}\right)=\text{Log}\;K+\left(\frac{1}{n}\right)\text{Log}\;C \end{array}$$

was used to evaluate the results. It is expressed in terms of soil equilibrium concentration, *x*/*m* (in* µ*g/g), adsorption parameter, “*K*” (in mL/g), and adsorption parameter, “*n*” (dimensionless). The results of Freundlich isotherms were plotted in Figure 3, whereas the parameters were summarized in Table 2. The correlation coefficients for the linear relationship of (2) were fairly high. The *n*-values obtained were comparable with those reported in the literature, 1.00–1.70. Using the sulfate as counter ion shows monolinear fits, whereas the chloride counter ion produced bilinear fits for any concentration (of the counter ion) and for both soils (see Figure 3). The two linear portions of the curve were considered separately with different slopes.

On the basis of the above results it can be seen that the cadmium concentration in the alluvial soil is highly influenced by its chemical and physical properties that are relevant to the adsorption ability (mainly clay composition and surface area) [31]. Also, the low contents of the calcareous soil in clay, organic matter, and the relatively low surface area may explain the lower adsorption capacity of the soil toward cadmium.

The formation of charged or uncharged complexes might be the reason for the differences in the ability of both soils to adsorb cadmium with changing the counter ion. Figure 1 shows that, for the same cadmium ion concentration in solution, the amounts of adsorbed Cd are appreciably lower in chloride systems than that in Na_2_SO_4_ systems. This observation can be attributed to the formation of various complexes of Cd^2+^ with Cl^−^ ligands (like CdCl_2_°, CdCl_3_^−^, CdCl_4_^−2^, CdCl_5_^−3^, etc.) where the Cl^−^ ion may coordinate with cadmium ions and be treated as an inner sphere complex with the surface [32]. On the other hand, from the previous chemistry studies, Na_2_SO_4_ tends to form an outer sphere complex in the alkaline conditions [32]. This observation agrees well with previous theoretical [29] and experimental [32] studies of the electrolyte effect on cadmium adsorption.

Also, Using NaCl produced two energy level sites for Cd adsorption in the Freundlich isotherms (Figure 3 where bilinear isotherms are produced for NaCl systems). This may account for the competition between Na^+^ from NaCl and Cd^2+^, to occupy some specific sites of the soil, given the fact that Cl^−^ has a tendency to make complex ions with Cd^2+^ making Na^+^ free in solution [32]. On the other hand, SO_4_^2−^ has no known complexation coordination with cadmium making Na^+^ in Na_2_SO_4_ less available for adsorption. This explanation agrees with our experimental results that suggest that lower concentration of the electrolyte (which means lower concentration of sodium ions) has higher adsorption ability (see Figure 1).

Several soil constituents are able to adsorb Cd^2+^. However, the high contents of clay, silt, organic matter, iron and manganese oxides, and calcium carbonate have always high capacity for adsorbing cadmium. However, no simple linear relationship should be expected. For example, the alluvial soil of this study exhibited three times higher capacity than the calcareous soil (see Table 2), although it contained five times more clay and six times more CEC than the calcareous soil (see Table 1). The higher capacity of the alluvial soil for Cd^2+^ adsorption is likely due in parts to the formation of an organo-clay complex with a higher affinity for Cd^2+^ or to the formation of a clay-Cd, organo-Cd bridge that increased the adsorption capacity of the system.

### 3.2. Gibbs Free Energy of Adsorption

The free energy of cadmium adsorption was calculated under different conditions of counter ion concentration. In this method the fractional surface coverage, *θ*, was fixed at 5 × 10^−10^ meq of cadmium adsorbed per cm^2^ of the surface area to provide the rational basis of comparing the surface of the soil. *θ* was calculated from the values of *x*/*m* per gram taken from the adsorption isotherm at a specific level that reaches all the adsorption isotherms. Thereby the principle of this method depends on keeping constant *θ* value for the comparison between soils under their different experimental conditions. The corresponding values for *x*/*m* divided by surface area of the electrolyte will be taken to calculate the free energy of adsorption Δ*G*_*θ*_°.

The standard free energy of adsorption at fixed *θ*, Δ*G*_*θ*_°, is determined from the following equation:

(3) $$\begin{array}{c} \Delta G_{\theta }^{{}^{\circ}}=-RT\ln a_{\text{C}{\text{d}}_{\text{eq}}}=-RT\ln\left[m_{\text{C}{\text{d}}_{\text{eq}}}\cdot \gamma_{\text{C}{\text{d}}_{\text{eq}}}\right], \end{array}$$

where *m*_Cd_eq__, *γ*_Cd_eq__, and *a*_Cd_eq__ are the molality, activity coefficient, and activity of cadmium ions at equilibrium conditions, respectively. The activity coefficient, *γ*_Cd_eq__, was calculated using Debye-Huckel theory for each equilibrium concentration and specific ionic strength, which eventually leads to calculating the Cd activity, *a*_Cd_eq__, and so the free energy of adsorption Δ*G*_*θ*_°.

Table 3 represents the summary of the Gibbs free energy of adsorption. All values were negative indicating spontaneous processes. The stronger the cadmium binding to the soil, the more negative the value of Δ*G*_*θ*_°. In general the values of Δ*G*_*θ*_° for the calcareous soil are higher than those for alluvial soil. At the same time, as seen previously (in Section 3.1 and Table 2), the maximum adsorption for the calcareous soil toward Cd^2+^ was lower. This means that calcareous soil provided less number of adsorption sites, though it exhibited stronger binding surfaces for cadmium ions. This might be explained by the fact that the mineral composition of the calcareous soil (see Table 1) with higher mica and attapulgite contents is high in this soil. It has been found by others [33–35] that these clay minerals have higher tendency to adsorb cadmium ions.

## 4. Conclusions

It appears likely that the existence of NaCl results in complexation of chloride and competition of sodium ions, with Cd^2+^. This accounts for the decrease of the maximum adsorption of cadmium on soil and also reduces the bonding energy coefficients compared to Na_2_SO_4_ systems. Due to this behavior, cadmium adsorption in case of NaCl salt matrix was about three times less than for Na_2_SO_4_. This effect is important since relatively moderate (to high) concentration of NaCl in soil solution will greatly reduce the adsorption of Cd^2+^ on soil surface. Thereby relatively high cadmium ion concentrations in soil solutions will be maintained especially under surface irrigation conditions, which increase the probability for ground water pollution with this harmful and toxic metal.

## Figures and Tables

**Table 1:** Characteristics and mineralogical analysis of the experimental soils.

Parameters	Alluvial soil	Calcareous soil
Texture of <0.5 mm:		
% sand (0.02–0.5 mm)	12.10	62.70
% silt (0–20 µm)	31.60	25.60
% clay (2 µm)	56.30	11.70
% calcium carbonate	2.14	21.80
% organic matter	1.75	0.30
CEC (meq/100 g soil)	48.20	8.70
Specific surface area (m^2^/g) (O.P. method)	233.80	87.60
Cd content (µg/g) (hot 1 : 1 HNO_3_ for 6 hrs)	0.36	0.06
Mineralogical analysis		
% montmorillonite	51.50	14.20
% kaolinite	15.00	17.20
% mica	7.50	26.30
% vermiculite	5.00	—
% attapulgite	—	22.00
% quartz	4.00	4.00
% feldspar	3.00	3.50
% free oxides	14.00	12.80

**Table 2:** The Langmuir and Freundlich coefficients for the two examined soils under different conditions of salt matrix and concentration.

Soil type	Salt matrix	Salt matrix conc.	Langmuir parameters		$\frac{a\times 100}{\text{CEC}}$	Freundlich parameters	
			*a* (µg/g)	*b* (ml/µg)		*k* (ml/µg)	*n*
Alluvial	Na_2_SO_4_	0.05 N	544	0.232	2.05	16.51	1.270
		0.15 N	448	0.242	1.69	14.09	1.297
	NaCl	0.05 N	255	0.137	0.96	*k*_1_ 5.62 *k*_2_ 27.03	*n*_1_ 1.077 *n*_2_ 2.333
		0.15 N	198	0.190	0.75	*k*_1_ 5.21 *k*_2_ 13.74	*n*_1_ 1.296 *n*_2_ 2.188

Calcareous	Na_2_SO_4_	0.05 N	170	0.368	3.12	15.31	2.026
		0.15 N	163	0.272	2.99	12.92	1.984
	NaCl	0.05 N	116	0.294	2.13	*k*_1_ 5.63 *k*_2_ 17.93	*n*_1_ 1.591 *n*_2_ 2.494
		0.15 N	85	0.330	1.56	*k*_1_ 5.63 *k*_2_ 16.58	*n*_1_ 1.732 *n*_2_ 3.778

**Table 3:** Free energy of cadmium adsorption for the two examined soils under different conditions of salt matrix and concentration.

Salt	Concentration	Free energy Δ*G*° (KCal/mole)	
		Alluvial soil	Calcareous soil
NaCl	0.05 N	−6.70	−7.14
	0.15 N	−6.62	−6.91

Na_2_SO_4_	0.05 N	−7.59	−8.00
	0.15 N	−7.44	−7.72
